# Identification of the instrumental quality and the sensory difference between homemade cooked rice and aseptic‐packaged cooked rice

**DOI:** 10.1002/fsn3.3742

**Published:** 2025-04-18

**Authors:** Sun‐Choung Ahn, Taeeun Kwon

**Affiliations:** ^1^ Department of Bio‐food & Foodservice Industry Shinhan University Uijeongbu‐si Korea; ^2^ Department of Biochemical Engineering Seoil University Seoul Korea

**Keywords:** aseptic‐packaged cooked rice, consumer acceptance, homemade cooked rice, quality properties

## Abstract

Consumers often prefer homemade cooked rice (HMCR), which differs from aseptic‐packaged cooked rice (APCR). This study provides sensory and instrumental data for commercial APCR and discusses physicochemical factors that affect sensory differences in commercial APCR and HMCR. The color revealed that yellowness and redness were influenced by rice embryos. Additionally, the poor appearance preference was associated with low grain wholesomeness, with a strong negative correlation between grain wholesomeness and appearance quality in cooked rice. The textural properties varied among samples, but there were no significant differences in texture preference. A major difference between HMCR and APCR was observed in the aroma profile. HMCR exhibited excellent aroma preference, particularly in terms of roasted and sweet flavors, which were key factors in aroma preferences. However, the presence of green and fatty flavor compounds negatively impacted the aroma quality. Conversely, roasted flavor compounds such as 2‐methoxy‐4‐vinylphenol positively contributed to the aroma quality of cooked rice. To improve consumer preference for APCR, efforts should focus on reducing green and fatty flavors while enhancing roasted flavors. By adjusting the flavor profile, the aroma quality of APCR can be enhanced, leading to greater consumer satisfaction.

## Graphical abstract

The aroma profile was an important factor in sensory properties between commercial and homemade cooked rice. The roasted flavor had a positive effect and the green and fatty flavor had a negative effect on the aroma quality of cooked rice. If the desired flavor can be added, it is expected that the consumer preference for aseptically packaged rice will improve.

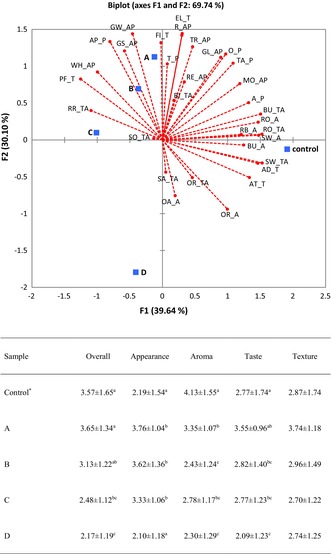

## 1 INTRODUCTION

Rice is a widely consumed food in the global food supply, accounting for approximately 35% of the world's food consumption, particularly as a staple in Asian countries. It is the most important staple food in the Korean diet. Korean rice (*Oryza sativa* L.) is mainly of the *japonica* variety, known for its round, thick, sticky, and glutinous characteristics compared to *indica* varieties. However, rice consumption has faced a decline owing to changing dietary habits, becoming a major concern in Korea (Kwak et al., 2015). The consumption of rice per capita dropped from 72.8 kg in 2010 to 61.8 kg in 2016. In contrast, the market for processed rice, such as the home meal replacement (HMR), has witnessed a rise. The grain consumption survey reported a 7.4% increase in rice used for processing from 2015 to 2016, primarily for HMR preparation (Statistics Korea, 2017). The rise in HMR consumption can be attributed to social changes, including an increase in smaller families consisting of greater female workforce participation, leading to a preference for convenient, ready‐to‐eat options (Kim et al., 1987; Lee, 2003). Various processed rice types, such as canned rice, retort rice, chilled rice, frozen rice, and aseptic‐packaged cooked rice (APCR) have been developed to meet consumer needs (Kim et al., 1987; Koh & Park, 1990). Among these, APCR stands out for its capability to maintain quality and freshness at room temperature for extended periods due to its production under clean and sterile conditions. The convenience factor, as it can be served in just 2–3 min via microwave heating, has contributed to its substantial sales growth in Korea, increasing from $7 million in 1997 to $120 million in 2008 (Kum, 2010).

Commercially available convenience rice such as retorted, frozen, or aseptic‐packaged rice suffers from sensory deficiencies compared to homemade cooked rice (HMCR; Yu et al., 2017). Despite the rapid growth in the processed cooked rice market, research on APCR remains limited (Kwak et al., 2015). Particularly in Korea, the focus has been on developing rice varieties suitable for processing such as Sungkwang, Hongkukgyeong, Goami, and Juan to promote rice consumption. Although Japanese varieties like Chu‐cheong and Koshihikari were initially used for APCR, the Juan variety has gained popularity since 2010 due to its successful restoration even after reheating with a microwave oven (Won & Oh, 2014). Proper reheating is critical for maintaining the texture and overall quality of APCR, as starch‐based foods like rice are challenging to restore once aged (Okabe, 1979; Rousset et al., 1999).

Overall consumer acceptance ratings for APCR in accordance with overall acceptance, appearance, aroma, and texture are significantly different compared to those for HMCR (Kwak et al., 2013). The taste quality of cooked rice significantly influences overall preference (Bett‐Garber et al., 2001; Champagne et al., 2004; Lyon et al., 1999; Meullenet et al., 1999). While several instruments are commercially available for rice taste evaluation, their correlation with overall flavors as determined by sensory test of rice has been reported to be low, indicating that these instruments may not accurately estimate rice taste evaluation value (Kawamura et al., 1996). Accurately measuring the preference for cooked rice has proven challenging due to subtle differences arising from various factors (Champagne et al., 1998; Hirannaiah et al., 2001; Leelayuthsoontor & Thipayarat, 2006; Meullenet et al., 2009; Mohapatra & Bal, 2006; Sesmat & Meullenet, 2001; Yau & Huang, 1996). Therefore, this study finds out the difference between APCR and HMCR by comparing the sensory of commercially available APCR and HMCR. By focusing on the sensory characteristics of the two rice types and the factors contributing to these characteristics, this study aims to shed light on the differences in sensory properties between HMCR and APCR while identifying the physicochemical factors contributing to these characteristics. Understanding these distinctions and identifying the underlying physicochemical factors will contribute valuable insights into the processed rice market and help meet consumers' preferences and expectations.

## 2 MATERIALS AND METHODS

### 2.1 Materials and reagents

Four commercial APCRs (samples A, B, C, and D) were selected based on sales volume and purchased from a local market in Korea. The rice (*O. sativa* L.) harvested at the Anseong farm (Gyeonggi‐do, Korea) in the fall of 2019 was used as control cooked rice. All chemicals and reagents were analytical grade and purchased from either Sigma‐Aldrich Co. or Daejung Chem. Deionized water (D‐water, i.e., resistance; 18.3 mΩ) was used in this study.

### 2.2 Preparation of cooked rice samples

The control cooked rice was prepared using a rice cooker (CRP‐HSXB0630FB, Cuckoo Electronics, Korea), following a typical Korean cooking method. The rice was washed with tap water, and the washing process was repeated three times. The washed rice was soaked in water for 25 min to improve its quality. After soaking, the water was drained, and 1.2 times the volume of water compared to rice was added to the soaked rice. The soaked rice and the additional water were placed into the rice cooker and the automatic cooking option was applied for approximately 15 min. The cooking was complete, and the rice was allowed to rest in the rice cooker for 10 min. In the rice cooker, the cooked rice was gently mixed excluding the bottom portion. The cooked rice was transferred to a bowl and served to the panelists for sensory evaluation. The commercial APCR was heated for 2 min using a microwave at 700 W (MS23K, Samsung Electronics Co. Ltd.) before being served to the panelists. Immediately after the cooking process, all cooked rice samples were stored at −80°C until the analysis of volatile flavor components.

### 2.3 Instrumental analysis of cooked rice

#### 2.3.1 Color

Colors of rice were measured by Hunter's colorimetric analysis method using a color difference meter (CR‐10 Plus, Minolta Co. Ltd.). Lightness (*L*, white +100 ~ 0 black), redness (*a*, red +100 ~ −80 green), and yellowness (*b*, yellow +70 ~ −80 blue) were measured, and overall color difference was calculated using the formula: $\mathrm{\Delta E}=\sqrt{{\left(\mathrm{\Delta L}\right)}^{2}+{\left(\mathrm{\Delta a}\right)}^{2}+{\left(\mathrm{\Delta b}\right)}^{2}}$.

#### 2.3.2 Texture

Texture profile analysis (TPA) was carried out using a texture analyzer (TMS‐Pro, Food Technology Co.) with a stainless steel probe with a diameter of 5 mm. Samples were placed in the center of the platform, and the calibrated probe was pressed down two‐bite compression by using a 5 kg load cell. It was compressed 3 mm with a time interval of 5 s at a speed of 0.5 mm/s. Textural properties were calculated in Texture Lab Pro (version 1.13‐002).

#### 2.3.3 Volatile flavor components

One gram of the sample was placed into a 2‐mL glass vial. Water was added to the sample by spraying it onto the top of the rice. Samples were preheated for 25 min at 80°C before sampling. The volatile components were collected by the headspace solid‐phase microextraction (SPME) method. The SPME fiber employed was 1 cm long and coated with 30/50 μm carboxen/divinylbenzene/polydimethylsiloxane (CAR/DVB/PDMS, Supelco). The volatile components of cooked rice were adsorbed for 15 min at 80°C at which the sample was shaken. The GC separations were achieved on Thermo Fisher Scientific TRACE™ 1310 Gas Chromatograph with TSQ™ 8000 GC‐MS. The GC was equipped with a capillary column (Agilent J&W Scientific, VF‐624 MS, 60 m × 0.25 mm × 1.4 μm). Helium was employed as the carrier gas. Samples were desorbed for 10 min in the injection port. The injector temperature was 270°C and the initial oven temperature was 40°C, which was held for 3 min. The oven was ramped at 5°C/min to 270°C. The final temperature was held for 10 min. The mass selective detector was operated in a scan mode from *m*/*z* 50 to 550.

### 2.4 Sensory evaluations of cooked rice

The sensory preference of cooked rice was assessed in terms of appearance, flavor, texture, and overall acceptability. The flavor was evaluated by separating the aroma and taste. The panel consisted of 30 undergraduate students in the Department of Food Science and Culinary Arts at Shinhan University, Gyonggi‐do, Korea. The preference ratings were recorded on a 5‐point scale, ranging from 1 (*dislike very much*) to 5 (*like very good*). For each aspect of appearance, aroma, taste, and texture, various attributes were further divided (Table 5). To evaluate the intensity of each attribute, 10 trained panelists, who had undergone 3 months of basic training and were experienced in the evaluation of cooked rice preference, were involved. The attribute intensity was quantified using a 1 to 9 scale (*very poor* to *very good*). To determine the intensity of each attribute and preference for cooked rice, a set of rice samples was presented randomly to the panel. Approximately 30 g of each sample was served in a white rice bowl and disposable spoons were provided to each panelist. Panelists were also instructed to rinse their mouths with bottled water (Pure Life, Nestle Co. Ltd.) before and between serving the sample. Paper cups and spoons were changed for each sample to avoid cross‐contamination and ensure accurate evaluation.

### 2.5 Statistical analysis

The means and standard deviations were calculated. The results were analyzed by the analysis of variance (ANOVA) method using SPSS for Windows, version 20.0 (SPSS, Inc.). A significant difference was defined as *p* < .05. In addition, Duncan's multiple range test was conducted to confirm the difference in sample characteristics when there was a significant difference between the samples. XLSTAT (version 2012; Addinsoft) was used for the entire multivariate analysis. Principle component analysis (PCA) was conducted to investigate correlations between the samples and sensory attributes.

## 3 RESULTS AND DISCUSSION

### 3.1 Instrumental analysis of cooked rice

#### 3.1.1 Color

The color attributes of APCR were evaluated using Hunter's values, and the result is summarized in Table 1. Among the samples, the control (HMCR) exhibited the lowest lightness (*L*), while no significant difference in lightness was observed among the commercial samples. The *L* values for the control and samples A, B, C, and D rice were 65.9, 68.4, 68.9, 67.4, and 68.2, respectively. All samples showed negative redness (*a*), indicative of green color perception. The sample C had the highest redness, while the control showed the highest greenness. The yellowness (*b*) values were positive for all samples, with sample C having the highest value (5.7) and sample D the lowest (4.7).

**TABLE 1:** Color of aseptic‐packaged cooked rice.

Hunter's color value	Rice samples				
	Control*	*A*	*B*	*C*	*D*
*L* **	65.9 ± 0.6	68.4 ± 0.8	68.9 ± 1.9	67.4 ± 1.0	68.2 ± 1.1
*a* ***	−2.2 ± 0.0^a^	−2.0 ± 0.0^b^	−1.9 ± 0.1^b^	−1.5 ± 0.1^c^	−1.9 ± 0.1^b^
*b* ****	5.7 ± 0.2^a^	5.4 ± 0.4^a^	5.7 ± 0.6^a^	6.9 ± 0.5^b^	4.7 ± 0.2^a^
Color difference (Δ*E*)*****	66.2 ± 0.7	68.6 ± 0.7	69.2 ± 1.8	67.8 ± 1.0	68.4 ± 1.1

*Note*: Values are shown as mean ± SD; *n* = 5.

Different superscript letters in a column indicate significant differences (*p* < .05).

* Control is homemade rice.

** Lightness (*L*): white +100 ~ 0 black.

*** Redness (*a*): red +100 ~ −80 green.

**** Yellowness (*b*): yellow +70 ~ −80 blue.

***** Overall color difference was calculated using a formula: $\mathrm{\Delta E}=\sqrt{{\left(\mathrm{\Delta L}\right)}^{2}+{\left(\mathrm{\Delta a}\right)}^{2}+{\left(\mathrm{\Delta b}\right)}^{2}}$.

#### 3.1.2 Texture

The hardness and springiness of all samples did not show a significant difference (Table 2). However, adhesiveness and cohesiveness showed a significant difference between the control and the APCR sample. The control exhibited the lowest adhesiveness and highest ratio of adhesiveness and hardness (A/H; 0.247) compared to the commercial samples. A higher ratio of A/H is indicative of better quality cooked rice (Okabe, 1979).

**TABLE 2:** Textural properties of aseptic‐packaged cooked rice.

Sample	Texture profile analysis (TPA)						
	Hardness (N)	Stickiness	Adhesiveness (N.sec)	Cohesiveness	Springiness (mm)	Chewiness (J)	A/H**
Control*	10,580 ± 800	−1390 ± 306^a^	−2617 ± 202^a^	2.36 ± 0.42^a^	1.02 ± 0.03	91,325 ± 2000^a^	0.247
*A*	10,585 ± 1069	−897 ± 170^ab^	−843 ± 85^b^	3.64 ± 0.50^b^	0.66 ± 0.58	133,336 ± 25,891^a^	0.080
*B*	9984 ± 300	−874 ± 249^ab^	−569 ± 61^b^	6.10 ± 0.28^c^	0.41 ± 0.72	449,374 ± 56,569^a^	0.057
*C*	9405 ± 1238	−1084 ± 385^a^	−1078 ± 780^b^	3.80 ± 0.21^b^	0.50 ± 0.70	149,260 ± 35,355^b^	0.115
*D*	8911 ± 557	−522 ± 152^b^	−422 ± 118^b^	3.85 ± 0.14^b^	0.53 ± 0.75	164,518 ± 134,789^a^	0.047

*Note*: Values are shown as mean ± SD; *n* = 3.

Different superscript letters in a column indicate significant differences (*p* < .05).

* Control is homemade rice.

** A/H is adhesiveness/hardness.

#### 3.1.3 Volatile flavor components

**FIGURE 1:**
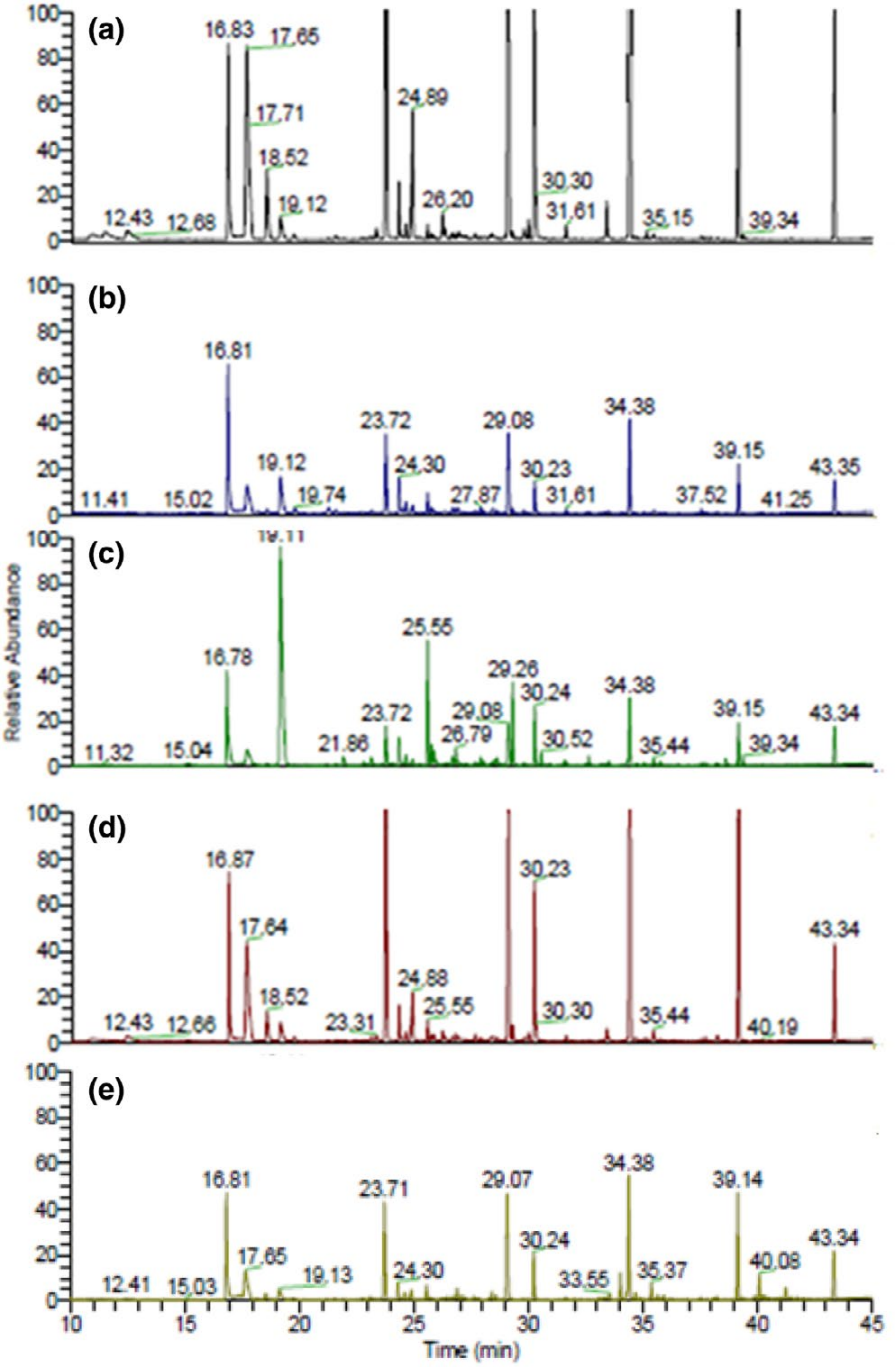
Total ion current chromatograms for the volatile compounds of cooked rice. (a–d) Aseptic‐packaged cooked rice. (e) Homemade cooked rice (control).

Rice is generally consumed after cooking, so its flavor is a crucial factor influencing the quality of cooked rice. The aroma is evaluated as the highest desired trait, followed by taste (Zhou et al., 2002). Furthermore, aroma is one of the primary sensory reference points, which is the most intuitive way for people to judge cooked rice (Zheng et al., 2022). The flavor is representative of their chemical profile based on their impact odorants. The volatile flavor components were analyzed using total ion current mass chromatograms, and the results are shown in Figure 1 and Table 3. It showed the volatile components in the HMCR (control) and four commercial APCRs. Samples C and D exhibited higher levels of green notes such as hexanal and 2‐pentyl‐furan compared to the control. The hexanal, 2‐pentyl‐furan, 2‐octenal, and 2‐nonenal in volatile compounds of sample C were about 7, 3, 25, and 2.7 times that of the control, respectively. Additionally, 2,4‐decadienal and 1‐octen‐3‐ol were detected in all APCR samples but absent in the control (HMCR). Hexanal, 2‐pentyl‐furan, 2‐nonenal, and 2,4‐decadienal are green notes. The green flavor seems to be due to the highest intensity of raw rice taste in sample C (Table 5). 2‐Octanal was a large amount contained in sample C. 2,4‐Decadienal and 2‐octanal are volatile chemicals with a fatty flavor character. Fatty flavor characteristics were undesirable in cooked rice because they are related to the rancidity of the lipid derived from rice embryo or bran. Especially, 2‐nonenal, 2,4‐decadienal, and octenal have low odor thresholds. The control had the highest concentration of 2‐methoxy‐4‐vinylphenol, contributing to its smoky and roasted aroma preference. Therefore, greenish and fatty flavors are a negative effect, but the roasted flavor is a positive effect on the aroma preference of cooked rice.

**TABLE 3:** Flavor volatiles identified in aseptic‐packaged cooked rice.

Compounds name	Flavor description	Threshold (PPb)	Area%				
			Control*	*A*	*B*	*C*	*D*
Aldehyde							
Pentanal	Fermented, winey, bready	1500		0.21	0.44	0.46	0.34
Hexanal	Green, vegetable, grassy, fresh	16–76	4.93	5.04	18.24	35.96	7.30
Heptanal	Green, aldehydic, fatty, oily grassy, fresh, herbal	3–21	1.01	0.53	1.24	1.04	1.28
2‐Heptenal, (Z)‐		13–80		0.86	1.84	2.36	1.60
Octanal	Aldehydic, citrus, orange, peel	0.7–45	0.78	0.70	1.43	1.74	1.72
2‐Octenal	Fatty, green, herbal	3–90	0.32	0.75	1.39	8.23	3.53
2‐Decenal, (E)‐	Waxy, fatty, earthy, coriander, green, mushroom, pork, fatty	0.3–230		0.02	0.27		0.48
2‐Nonenal, (E)‐	Green, cucumber, aldehydic, fatty	0.08–6	0.36	0.16	0.73	0.97	0.41
2,4‐Decadienal, (E,E)‐	Fatty, chicken, green	0.07		0.41	0.56	0.31	0.99
2‐Octenal, 2‐butyl‐						0.81	
Dodecanal	Soapy, waxy, aldehydic, citrus, orange	1–2		0.74		0.88	
Ketone							
3‐Octen‐2‐one	Earthy, oily, mushroom					0.32	
Alcohol							
1‐Octen‐3‐ol	Mushroom, earthy, fungal, green, oily, vegetable	1		0.51	0.86	1.58	1.62
1‐Hexanol, 2‐ethyl‐	Citrus, sweet, fatty, oily		1.00	1.03	0.98	0.34	1.67
Hydrocarbon							
Dodecane	Alkane		3.13		1.60	0.33	1.06
Undecane			2.58	1.40	2.13		1.79
1‐Dodecene						0.42	
Dodecane	Alkane		0.82	2.24	1.55	0.37	1.59
Heptadecane			5.06				
Tetradecane	Mild waxy		0.84	0.53	1.68	0.33	0.62
Aromatic derivatives							
Benzene, ethyl‐				0.47	2.97		
Benzene, 1,3‐dimethyl‐	Plastic		0.53	1.04	2.06	0.22	0.57
Benzaldehyde	Fruity, sweet, almond, cherry, nutty	350–3500	0.77	1.39	1.90	1.07	1.36
Benzaldehyde, 2,5‐dimethyl‐				1.01	1.79	0.94	3.08
2‐Methoxy‐4‐vinylphenol	Smoky, woody, bacon, roasted, peanut, ceder	3	1.18	0.40	0.42		1.05
Alkylfuran derivatives							
Furan, 2‐butyl‐	Fruity, sweet, wine‐like, spicy					1.43	
Furan, 2‐pentyl‐	Green, fruity		4.49	2.40	7.28	14.97	5.88
Terpene							
1R‐à‐Pinene	Herbal, terpenic, aromatic, minty	6	0.29	2.45			1.83
dl‐Limonene	Citrus, herbal, terpenic, camphoraceous	10		1.17			

* Control is homemade cooked rice.

### 3.2 Sensory evaluations of cooked rice

The sensory evaluations and consumer acceptance results provide valuable insight into the preferences and attributes of cooked rice samples (Tables 4 and 5). Sample A showed the highest overall acceptability, with the control ranking second, though not significantly different. However, both the control and sample D received relatively low for appearance preference, with the control being higher than sample D in overall acceptability. The biggest difference between the control and sample D was aroma preference. The aroma of cooked rice largely determines the taste quality of cooked rice (Zheng et al., 2022). The higher aroma preference in the control (HMCR) could be attributed to its intensified roasted and sweet aromas. These flavors likely contribute to the overall preference of the control (HMCR), explaining its higher overall acceptability. The preference for appearance was relatively low for both the control (HMCR) and sample D, possibly due to factors such as low grain wholesomeness. Grain wholesomeness was negatively correlated with appearance preference, while white color and grain size were positively correlated (Goodwin jr. et al, 1992; Kim & Kim, 2007; Suwansr et al., 2002). The study revealed that white color, grain wholesomeness, and the size of cooked rice significantly influenced appearance, rather than factors like glossiness, moisture, and transparency. The intensity of the rice embryo was the highest in sample C and the *b* value (yellowness) of sample C was also the highest (Table 1). This suggests that the rice embryo significantly influenced both redness and yellowness, particularly impacting the yellowness. Furthermore, the *b* value (yellowness) was highly negatively correlated with rice quality (Chung, 2002), indicating that higher yellowness may be associated with lower rice quality. The taste preference for sample A was the highest, though not significantly different from the control and samples B and C. The texture preference was ranked in the order of A, B, control, C, and D, but no significant differences were observed between samples. The sensory attribute of adhesiveness was the highest in control. It was related to the lowest adhesiveness of the control in TPA (Table 2). The rice samples differed (*p* < .05) with retrospect to the sensory attribute (in order of *F* value) glossiness, grain wholesomeness, adhesiveness, moisture, grain size, roughness, sweet aroma, roasted aroma, transparency, roasted taste, rice embryos, white color, sweet taste, and raw rice taste. No significant difference was noted for rice bran aroma, old rice aroma, burnt aroma, off aroma, salty taste, bitter taste, sour taste, old rice taste, firmness, elasticity, particle feeling, and after‐taste.

**TABLE 4:** Acceptance of overall, appearance, aroma, and texture for aseptic‐packaged cooked rice.

Sample	Overall	Appearance	Aroma	Taste	Texture
Control*	3.57 ± 1.65^a^	2.19 ± 1.54^a^	4.13 ± 1.55^a^	2.77 ± 1.74^a^	2.87 ± 1.74
*A*	3.65 ± 1.34^a^	3.76 ± 1.04^b^	3.35 ± 1.07^b^	3.55 ± 0.96^ab^	3.74 ± 1.18
*B*	3.13 ± 1.22^ab^	3.62 ± 1.36^b^	2.43 ± 1.24^c^	2.82 ± 1.40^bc^	2.96 ± 1.49
*C*	2.48 ± 1.12^bc^	3.33 ± 1.06^b^	2.78 ± 1.17^bc^	2.77 ± 1.23^bc^	2.70 ± 1.22
*D*	2.17 ± 1.19^c^	2.10 ± 1.18^a^	2.30 ± 1.29^c^	2.09 ± 1.23^c^	2.74 ± 1.25

*Note*: Acceptance ratings scores reflect the mean ± SD on a 5‐point scale; 1 – dislike very much, 2 – dislike, 3 – fair, 4 – like, 5 – like very good.

Different superscript letters in a column indicate significant differences (*p* < .05).

* Control is homemade cooked rice.

**TABLE 5:** Twenty‐seven sensory attributes intensity of aseptic‐packaged cooked rice.

Modality	Attribute	Control*	*A*	*B*	*C*	*D*	*F* value	Sig.
Appearance	Glossiness	7.00 ± 2.88^a^	5.37 ± 1.67^b^	6.70 ± 1.90^a^	4.41 ± 2.14^b^	2.33 ± 1.92^c^	21.140	<0.001
	Moisture	6.85 ± 2.77^a^	5.07 ± 1.80^bc^	6.19 ± 2.24^ab^	3.89 ± 1.87^cd^	3.59 ± 2.59^d^	10.326	<0.001
	Transparency	5.37 ± 3.55^a^	6.26 ± 1.68^a^	6.33 ± 2.60^ab^	3.96 ± 1.79^b^	3.74 ± 2.16^b^	6.818	<0.001
	White color	3.67 ± 3.28^a^	5.89 ± 2.90^a^	4.63 ± 2.88^ab^	5.22 ± 2.38^ab^	4.26 ± 2.09^b^	2.653	0.036
	Grain wholesomeness	4.48 ± 2.64^a^	6.11 ± 2.17^b^	6.33 ± 2.29^b^	6.15 ± 2.16^b^	2.93 ± 2.11^c^	11.284	<0.001
	Rice embryos	5.15 ± 2.77^ab^	4.00 ± 2.14^bc^	4.54 ± 2.61^ab^	5.46 ± 2.35^a^	2.96 ± 1.80^c^	4.605	0.002
	Roughness	5.81 ± 2.95^ab^	6.04 ± 2.24^ab^	6.85 ± 1.92^b^	5.30 ± 2.13^a^	3.44 ± 2.24^c^	8.116	<0.001
	Size of cooked rice	4.63 ± 2.66^ab^	5.48 ± 1.91^bc^	6.93 ± 2.45^d^	6.48 ± 2.12^cd^	3.44 ± 2.31^a^	10.071	<0.001
Aroma	Roast	6.70 ± 2.76^a^	4.04 ± 2.56^b^	4.37 ± 2.53^b^	3.89 ± 2.20^b^	3.48 ± 2.28^b^	7.345	<0.001
	Sweet	6.56 ± 2.50^a^	3.59 ± 2.41^b^	3.89 ± 2.62^b^	3.67 ± 1.84^b^	3.37 ± 2.66^b^	8.022	<0.001
	Rice bran	5.12 ± 3.44	3.46 ± 2.14	4.08 ± 2.67	4.08 ± 2.48	3.54 ± 2.30	1.619	0.173
	Old rice	4.54 ± 3.26	3.80 ± 2.60	3.85 ± 2.48	3.15 ± 2.19	4.62 ± 2.77	1.318	0.267
	Burnt	3.74 ± 3.29	2.33 ± 2.08	2.74 ± 2.57	2.85 ± 2.71	2.48 ± 2.64	1.130	0.345
	Offaroma	3.62 ± 3.24	3.31 ± 2.69	3.88 ± 2.75	3.31 ± 2.51	3.85 ± 3.06	0.248	0.910
Taste	Sweet	5.26 ± 2.88^a^	4.22 ± 2.92^ab^	3.67 ± 2.08^b^	3.30 ± 1.98^b^	4.26 ± 2.73^ab^	2.556	0.042
	Salty	2.07 ± 2.46	1.67 ± 1.47	1.81 ± 1.59	2.26 ± 2.55	1.89 ± 2.10	0.331	0.856
	Bitter	2.15 ± 1.97	2.31 ± 2.40	2.31 ± 2.53	2.00 ± 1.98	2.19 ± 2.26	0.084	0.987
	Sour	2.15 ± 2.13	1.77 ± 1.80	2.38 ± 2.17	2.27 ± 2.36	2.00 ± 1.98	0.341	0.850
	Roasted	6.19 ± 3.00^a^	4.41 ± 2.27^b^	3.52 ± 2.19^b^	3.41 ± 1.97^b^	3.74 ± 2.49^b^	6.195	<0.001
	Burnt	5.15 ± 3.23^a^	4.19 ± 2.50^ab^	3.74 ± 2.67^ab^	3.11 ± 2.08^b^	3.44 ± 2.41^b^	2.536	0.043
	Raw rice	2.78 ± 2.38	3.15 ± 2.14	3.19 ± 2.42	4.04 ± 2.90	2.93 ± 2.18	1.102	0.358
	Old rice	4.04 ± 3.20	3.56 ± 2.29	2.78 ± 1.95	4.04 ± 2.17	3.67 ± 2.54	1.176	0.324
Texture	Firmness	4.41 ± 2.65	5.48 ± 2.21	4.26 ± 2.55	4.70 ± 2.20	3.59 ± 2.41	2.203	0.072
	Elasticity	4.41 ± 2.27	5.00 ± 2.42	4.48 ± 2.64	4.19 ± 2.09	3.74 ± 2.55	0.983	0.419
	Adhesiveness	7.44 ± 2.41^a^	4.78 ± 2.03^bc^	4.59 ± 2.15^bc^	3.81 ± 1.86^c^	5.15 ± 2.66^b^	10.419	<0.001
	Particle feeling	3.59 ± 2.71	4.33 ± 2.29	4.63 ± 2.29	4.70 ± 2.33	3.89 ± 3.11	0.941	0.442
	After taste	5.22 ± 2.44	4.56 ± 2.10	3.96 ± 1.79	3.81 ± 1.94	4.70 ± 2.76	1.777	0.137

*Note*: Sensory evaluation scores reflect the mean ± SD of each sensory attribute on a 9‐point scale; 1 – very poor, 3 – poor, 5 – fair, 7 – good, 9 – very good.

Different superscript letters in a column indicate significant differences (*p* < .05).

* Control is homemade cooked rice.

**FIGURE 2:**
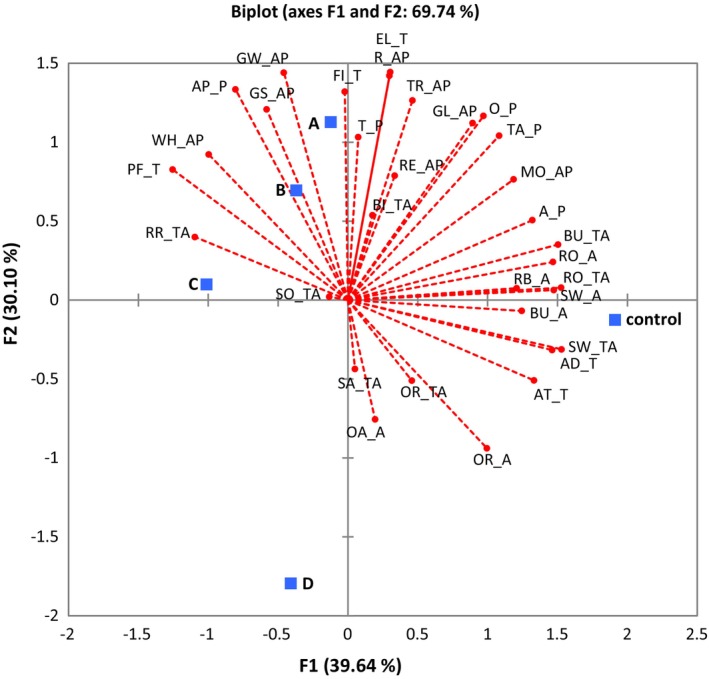
Principal components analysis (PCA) biplot for the sensory attribute of aseptic‐packaged cooked rice. Square represent different rice varieties (control, HMCR; a, APCR‐A; b, APCR‐B; c, APCR‐C; d, APCR‐D). Abbreviations for sensory attributes are as follows: GL, glossiness; MO, moisture; TR, transparency; WH; whiteness; GW, grain wholesomeness; RE, rice embryos; R; roughness; GS, grain size; RO, roasted; SW, sweet; RB, rice bran; OR; old rice; BU, burnt; OA, off aroma; SA, salty; BI, bitter; SO, sour; RR, raw rice; FI, firmness; EL, elasticity; AD, adhesiveness, PF, particle feeling; AT, after taste. Categories: AP, appearance; A, aroma; TA, taste; T, texture; P, preference.

The PCA biplot (Figure 2) revealed that aroma and taste attributes were associated with the first dimension (F1), while appearance attributes were related to the second dimension (F2). The biplot explained 69.74% of the total variations (F1: 39.64% and F2: 30.10%). The first dimension of the plot (F1) was based on adhesiveness, roast taste, burnt taste, sweet aroma, roast aroma, sweet taste, after taste, aroma preference, burnt aroma, rice bran aroma, moisture, taste preference, and old rice aroma on the right, and particle feeling, raw rice taste, whiteness on the left. The second dimension (F2) was characterized by elasticity, grain wholesomeness, roughness, appearance preference, firmness, transparency, grain size, overall preference, and glossiness on the positive side. F1 had characteristics related to aroma and taste, and F2 had characteristics related to appearance. Sample D is the furthest away from the center of the biplot. The control (HMCR) displayed flavor and taste characteristics, while samples A and B were characterized by appearance and texture attributes. Sample C exhibited attributes of raw rice taste and sour taste, and sample D had a salty taste and off‐flavor characteristic, which contributed to its low overall acceptability.

## 4 CONCLUSIONS

The aroma preference played a significant role in the overall preference for the APCR. While the textural properties differed among the samples, no significant differences in texture preference were observed. The overall preference for HMCR and sample A were the highest, but their characteristics differed. Sample A was associated with appearance and texture attributes, while the HMCR was linked to taste and aroma attributes. Especially, roasted and sweet flavors were the most prominent differences between HMCR and APCR. This study also found that the greenish and fatty flavor had a negative effect on aroma preference, while the roasted flavor had a positive effect on the aroma preference of cooked rice. Furthermore, 2‐methoxy‐4‐vinylphenol, contributing to smoky and roasted aroma preference, was identified as a key odorant compound in HMCR.

## AUTHOR CONTRIBUTIONS

**Sun‐Choung Ahn:** Conceptualization (equal); data curation (supporting); formal analysis (supporting); funding acquisition (lead); investigation (equal); methodology (supporting); project administration (equal); supervision (equal); validation (supporting); visualization (supporting); writing – review and editing (equal). **Taeeun Kwon:** Conceptualization (equal); data curation (lead); formal analysis (lead); investigation (equal); methodology (lead); project administration (equal); supervision (equal); validation (equal); visualization (lead); writing – original draft (lead); writing – review and editing (equal).

## CONFLICT OF INTEREST STATEMENT

The authors declare that they have no known competing financial interests or personal relationships that could have appeared to influence the work reported in this article.

## Data Availability

The data that support the findings of this study are available on request from the corresponding author.
